# 

**Published:** 1913-12

**Authors:** 


### CONTENTS.

ORIGINAL COMMUNICATIONS—

Georgia Surgeons' Club, Dr. Geo. H. Noble, Gynecological

Dr. James N. Ellis, Surgical Clinic at the Grady Hospital. November 5th, 1913 .......... 387

Clinic at the Grady Hospital, Atlanta, Ga.......... 383

Clinic Held for the Georgia Surgeons' Club at Wesley Memorial Hospital, Nov. 5, 1913. By Dr. Willis Jones, .......... 393

Symposium—"The Necessity of an Early Exploratory Incision in Obscure Abdominal Conditions".......... 401

Value of Scarlet Red Ointment in Granulating Wounds. By T. C. Davison, M. D., Atlanta, Ga.......... 408

Genito-Urinary Clinic—Atlanta Medical College, 11 A. M. Thursday. By Prof. A. L. Fowler; Dr. J. Calvin Weaver, Associate; Dr. T. B. Armstrong, Anaesthetist. .......... 410

Dr. L. C. Fischer, Atlanta Surgical Clinic, at the Davis & Fischer Sanatorium .......... 414

Demonstration Before the Georgia Surgeons' Club. By Dr. W. F. Shallenberger and Dr. Montague L. Boyd. .417

Air to Tuberculosis." .......... 420

EDITORIAL .......... 421

SELECTIONS AND ABSTRACTS .......... 423

BOOK REVIEWS .......... 429

